# Local Drug Delivery for Prevention of Hearing Loss

**DOI:** 10.3389/fncel.2019.00300

**Published:** 2019-07-09

**Authors:** Leonard P. Rybak, Asmita Dhukhwa, Debashree Mukherjea, Vickram Ramkumar

**Affiliations:** ^1^Department of Otolaryngology, School of Medicine, Southern Illinois University, Springfield, IL, United States; ^2^Department of Pharmacology, School of Medicine, Southern Illinois University, Springfield, IL, United States

**Keywords:** intra-tympanic injection, cisplatin, noise, hearing loss, acoustic trauma, ototoxicity

## Abstract

Systemic delivery of therapeutics for targeting the cochlea to prevent or treat hearing loss is challenging. Systemic drugs have to cross the blood-labyrinth barrier (BLB). BLB can significantly prevent effective penetration of drugs in appropriate concentrations to protect against hearing loss caused by inflammation, ototoxic drugs, or acoustic trauma. This obstacle may be obviated by local administration of protective agents. This route can deliver higher concentration of drug compared to systemic application and preclude systemic side effects. Protective agents have been administered by intra-tympanic injection in numerous preclinical studies. Drugs such as steroids, etanercept, D and L-methionine, pifithrin-alpha, adenosine agonists, melatonin, kenpaullone (a cyclin-dependent kinase 2 (CDK2) inhibitor) have been reported to show efficacy against cisplatin ototoxicity in animal models. Several siRNAs have been shown to ameliorate cisplatin ototoxicity when administered by intra-tympanic injection. The application of corticosteroids and a number of other drugs with adjuvants appears to enhance efficacy. Administration of siRNAs to knock down AMPK kinase, liver kinase B1 (LKB1) or G9a in the cochlea have been found to ameliorate noise-induced hearing loss. The local administration of these compounds appears to be effective in protecting the cochlea against damage from cisplatin or noise trauma. Furthermore the intra-tympanic route yields maximum protection in the basal turn of the cochlea which is most vulnerable to cisplatin ototoxicity and noise trauma. There appears to be very little transfer of these agents to the systemic circulation. This would avoid potential side effects including interference with anti-tumor efficacy of cisplatin. Nanotechnology offers strategies to effectively deliver protective agents to the cochlea. This review summarizes the pharmacology of local drug delivery by intra-tympanic injection to prevent hearing loss caused by cisplatin and noise exposure in animals. Future refinements in local protective agents provide exciting prospects for amelioration of hearing loss resulting from cisplatin or noise exposure.

## Introduction

### Blood-Labyrinth Barrier

A wide variety of drugs has been used to treat inner ear diseases. However, the efficacy of systemic drug therapy is frequently limited by restricted uptake into the inner ear by a barrier system, the blood labyrinth barrier (BLB). The BLB was a term that was developed from the fact that the inner ear fluids have a composition that is distinct from blood. These features provide a diffusion barrier that excludes many substances from entering the inner ear from the blood. Tracer studies demonstrated that various substances enter the perilymph slowly after systemic injection (Juhn et al., 1981). The rate of penetration into perilymph is generally inversely proportional to the molecular weight of compounds tested. This blood-perilymph barrier appears to be situated in the blood vessels located in the modiolus of the cochlea. Substances traveling in these vessels are transported from blood into perilymph of the scala tympani and scala vestibuli (Zou et al., 2016). The cochlear glomeruli of Schwalbe form vessel loops of capillaries adjacent to both perilymphatic scalae (Franz et al., 1993). This barrier consists of non-fenestrated capillaries with a continuous endothelial lining with tight junctions between endothelial cells.

The perilymph-endolymph barrier consists of tight junctions between cells lining Reissner’s membrane (Zou et al., 2016). Substances contained in perilymph may enter endolymph if they are able to penetrate this barrier.

**FIGURE 1:**
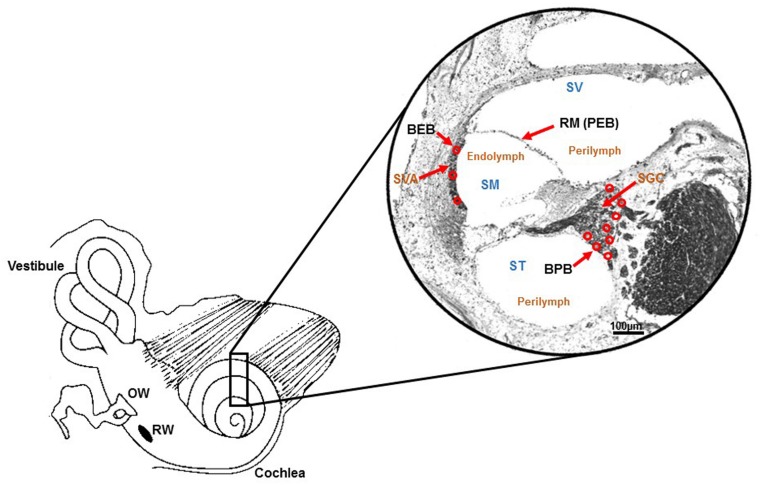
Schematic illustration of barriers within inner ear. Drawing of the cochlea and photomicrograph of a mid-modiolar section of the rat cochlea (stained with Sudan black) demonstrating the various barriers within the inner ear. These include the blood-endolymph barrier (BEB) in the stria vascularis; the blood-perilymph barrier (BPB); and the perilymph-endolymph barrier (PEB) which is formed by Reissner’s membrane (RM). Other abbreviations are: SVA (stria vascularis), SGC (spiral ganglion cells), SV (scala vestibuli), ST (scala tympani) and SM (scala media). Perilymph is contained within SV and ST, and endolymph is present in SM. Adapted from Zou et al. (2016).

The blood-endolymph barrier consists of endothelial cells of capillaries in the stria vascularis that separate the contents of blood in the capillary lumen from the interstitial fluid of the stria vascularis. These marginal cells have tight junctions between them that can restrict passage of substances from blood to endolymph. Within the stria vascularis, marginal cells and the basal cells comprise the intrastrial compartment which separates fluid in that compartment from endolymph in the scala media. This barrier is called the the intrastrial fluid-blood barrier (Shi, 2016). Additional components of the blood-strial barrier have been recently identified. These include pericytes and perivascular resident macrophage-type melanocytes. These three cell types: endothelial cells, pericytes and perivascular resident macrophages are connected by an extracellular basement membrane. Collectively, these cells together form a “cochlear-vascular unit” in the stria vascularis (Shi, 2016; Nyberg et al., 2019). Substances contained in the blood vessels of the stria vascularis may enter the intra-strial space through the blood-strial barrier and then gain access to the endolymph through the marginal cells lining scala media. The various inner ear barriers are illustrated in Figure 1.

**FIGURE 2:**
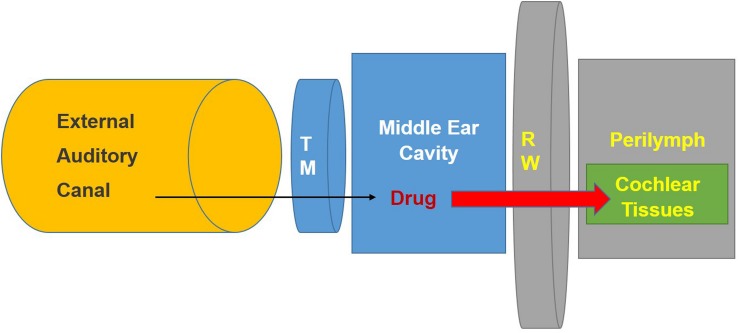
Schematic diagram illustrating method for intra-tympanic injection. The drug is injected through the tympanic membrane (Heinrich et al., 2016) into the middle ear. It then can penetrate the round window membrane (RW) to enter the inner ear fluids (perilymph) and tissues. Modified from the ACS article https://pubs.acs.org/doi/abs/10.1021%2Facs.jmedchem. 7b01653 with permission.

**FIGURE 3:**
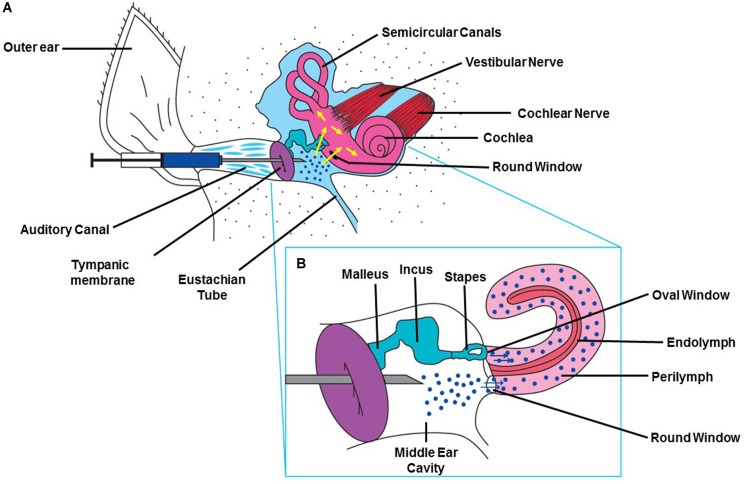
Method for intra-tympanic injection. **(A)** A syringe with an attached 30 gage needle ½ to 5/8th of an inch in length is directed through the external ear canal to the tympanic membrane of anesthetized animal with an operating microscope. A single puncture is made in the anterior inferior region. The desired solution is slowly injected into the middle ear and the rat is left undisturbed for 15 min with injected ear facing upward (Sheehan et al., 2018). **(B)** Drawing depicting the injected drug traversing the RWM and entering the cochlea. This figure was modified with permission from the image screenshot at 1:59 of the article https://www.jove.com/video/56564/trans-tympanic-drug-delivery-for-the-treatment-of-ototoxicity.

Treatments for hearing disorders such as cisplatin ototoxicity and noise-induced hearing loss could be administered systemically. However, this approach poses several difficulties: The potential protective agent may (1) not readily cross the BLB (size of the substance) and thus not reach its intended target cells in the cochlea in effective concentrations, (2) could interfere with the desired therapeutic effect of cisplatin (e.g., sodium thiosulfate), and (3) could cause off-target unwanted side effects, especially if high doses are needed to provide protection against hearing loss. One of these off-target effects could result in exacerbation of hearing loss instead of its amelioration. Local application by intra-tympanic administration is minimally invasive and allows drugs or other therapeutic agents to gain access to the inner ear with few or no systemic side effects and minimal risks of interference with the anti-tumor action of drugs like cisplatin. Intra-tympanic injection involves the instillation of substances through the tympanic membrane (Sheehan et al., 2018), shown schematically as a flowchart in (Figure 2) and in detail in (Figure 3). Another approach for localized drug delivery is through the bulla into the middle ear cavity or application of drugs directly to the round window membrane (RWM). This paper reviews preclinical strategies for local delivery of drugs by intra-tympanic injection to prevent hearing loss from cisplatin and noise trauma.

### Pharmacokinetics of Local Drug Delivery

Salt and Plontke have described the use of a standard pharmacologic acronym to describe the pharmacokinetics of drugs after intra-tympanic delivery to the cochlea (Salt and Plontke, 2009, 2018). This acronym is LADME. This term includes liberation, absorption, distribution, metabolism, and elimination of drugs applied intra-tympanically.

Liberation indicates the release of the agent from the dosage form administered to the tympanic cavity into the inner ear. The use of simple drug solutions may not provide sufficient duration of protection. Therefore, incorporation of the drug into controlled-release vehicles can prolong the presence of the drug in the middle ear cavity for transfer through the round or oval window membrane. A variety of technologies have been developed to allow extended release of drugs injected intra-tympanically. Some examples of these methods to prolong the release of active protective agents to the round or oval window membrane include:

• Implantation of an osmotic or digital mini pump to provide sustained delivery of various antioxidants to the RWM in guinea pigs (Wimmer et al., 2004).
• The development and utilization of OTO-104 which contains micronized dexamethasone 160 in poloxamer gel. Significant levels of dexamethasone were maintained in perilymph for 3 months in guinea pigs and more than 1 month in sheep (Piu et al., 2011).
• The intratympanic injection of a hyaluronic acid liposomal gel for sustained delivery of dexamethasone was tested in the guinea pig. The gel remained for a long time in the middle ear cavity and in the RWM after intra-tympanic injection without any evidence of ototoxicity. This resulted in sustained release of dexamethasone in perilymph for 1 month (El Kechai et al., 2016). This appears to be a promising way to deliver corticosteroids to the inner ear to provide sustained protection against cochlear insults.
• Dormer et al. have developed an innovative system to provide extended release of drugs for intra-tympanic injection (Dormer et al., 2019). It contains a film forming agent (FFA) and microspheres to provide prolonged delivery of betamethasone in a formulation called ORB-202 to the round window and inner ear in mice. This technology has shown that corticosteroids contained in microspheres with FFA were retained on the RWM for up to 5 weeks on necropsy examination. A recent review discusses various approaches to nanotechnology for inner ear applications. Only a few examples will be presented below.
• Li et al. have developed a nanohydrogel delivery system, which combines nanotechnology with a chitosan-glycerophosphate hydrogel delivery system. These nanoparticles could be delivered across the mouse RWM to reach structures in the scala media (Li et al., 2017).

Liberation can also result from drug generation in the middle ear from gene or cell therapy by which cells are enabled to produce a therapeutic agent (Salt and Plontke, 2018).

A variety of delivery paradigms has been tried to affect liberation. These include rates of injection with pumps, various other devices, and rates of elution among others (Salt and Plontke, 2018).

Absorption describes the passage of the drug from the middle ear cavity to the perilymph through the RWM, oval window or cochlear bone. The RWM in mammals comprises of 3 cellular layers: an epithelial layer that faces the middle ear space; a connective tissue layer in the middle; and a layer that faces the perilymph of the scala tympani (Goycoolea, 1992; Goycoolea and Lundman, 1997). The epithelial layer facing the middle ear cavity has tight junctions between cells (Salt and Plontke, 2009). It appears that the layers of the round window are involved in absorption and secretion of substances to and from the inner ear. Tracer molecules such as cationic ferritin, horseradish peroxidase, and 1 micron latex microspheres instilled into the middle ear pass through RWM, into the inner ear and have been detected in pinocytotic vesicles in the RWM (Goycoolea et al., 1988). Permeability of the RWM depends on molecular weight, solubility in lipids, concentration and charge, as well as the RWM thickness (Goycoolea and Lundman, 1997). Some examples of substances shuttled across RWM are:

• Nanoparticles are translocated across the RWM by endocytosis. This process follows three different mechanisms: macropinocytosis, caveolin-mediated endocytosis, and clathrin-mediated endocytosis (Zhang et al., 2018).
• A study of RW permeation enhancers was carried out using fluorescein tagged dexamethasone applied to the RW niche in guinea pigs. DMSO, N-methylpyrrolidone and benzyl alcohol provided significantly higher entry than that observed in controls (Li et al., 2018).
• Adjuvants were used to enhance the permeation of dexamethasone through the RWM. Application of dexamethasone on a hyaluronic acid sponge with or without histamine or dexamethasone with histamine provided greater penetration into perilymph in guinea pigs than did dexamethasone alone (Creber et al., 2019).
• A novel method to enhance the delivery of siRNA to the cochlea was developed using a recombination protein, double-stranded RNA-binding domains (TAT-DRBDs). The authors showed efficient siRNA transfection to the cochlea of the chinchilla with this delivery system. They were able to demonstrate successful transfection of Cy3-labeled siRNA into cells of the inner ear through the intact RWM, including the IHCs, OHCs, and vestibular cells in the crista ampullaris, macula utriculi, and macula sacculi (Qi et al., 2014).

The oval window and thin bone of the stapes footplate may provide other routes of entry for drugs applied intra-tympanically. Although the oval window has been shown to be permeable to horseradish peroxidase (Tanaka and Motomura, 1981), it is uncertain how much drug would enter by this route (Salt and Plontke, 2009) unless it were directly applied on the stapes footplate (King et al., 2011) or unless it were applied using nanoparticles. In the latter case, the application of fluorescent chitosan nanoparticles by intra-tympanic injection in guinea pigs was associated with penetration of both RWM and oval window, but with much stronger fluorescence in the vestibule than in the cochlea (Ding et al., 2019). On the other hand King et al. showed with MRI that most of the gadolinium applied through tympanic cavity entered perilymph through stapes (King et al., 2011). The bone of the otic capsule may provide a route of transport from the middle ear to the apical regions of the cochlea in guinea pigs (Salt and Plontke, 2009; Salt and Hirose, 2018).

Distribution includes the mechanism by which the drug is spread within and between perilymph and endolymph and how it passes from inner ear fluids into tissues of the cochlea. The distribution of drugs within the cochlear fluids proceeds primarily by passive diffusion but if fluid flow is present, volume flow can occur (Salt and Plontke, 2018). Distribution also includes flow of substances from fluid spaces to extracellular spaces of cochlear tissue compartments, especially in areas where cell layers are not complete or where cells do not possess tight junctions (Salt and Plontke, 2018). Distribution includes all forms of drug movement within the inner ear (Salt and Plontke, 2018). Distribution may be enhanced by the use of magnetic nanoparticles to cross the RWM and enter the inner ear fluids and tissues. The use of an external magnet can control the delivery of drug packaged in magnetic nanoparticles. After the magnet is taken away, the drug containing nanoparticles can then diffuse through perilymph. Fluorescent magnetic nanoparticles have been shown to traverse the RWM and gain access to the perilymph (Li et al., 2017).

Metabolism is the chemical alteration of drugs administered into the ear. It is also known as biotransformation (Salt and Plontke, 2018). Once a drug enters the inner ear, it can be broken down into substances that are more bioactive or that are inactivated. The intra-tympanic administration of liposomal hyaluronic acid led to the transformation of dexamethasone phosphate, a prodrug, into the active form, dexamethasone (El Kechai et al., 2016). Thus the metabolite may have a greater affinity for its receptors in the inner ear. Changes in physical characteristics of a drug resulting from metabolism can alter its ability to traverse cell membranes and layers in the inner ear. These changes can alter the rate of elimination (Salt and Plontke, 2018).

Elimination includes the processes by which the drug or its metabolites are transferred from the inner ear into other body fluids such as blood or cerebrospinal fluid or transport from the inner ear to the middle ear cavity. From the middle ear cavity a drug administered intra-tympanically can exit be eliminated via the Eustachian tube into the pharynx (Salt and Plontke, 2018). A novel approach to elimination of substances transported into the cochlea is to administer magnetic nanoparticles, then remove them using an external magnet. This has been demonstrated using fluorescently tagged magnetic nanoparticles *in vivo* (Li et al., 2018). Middle ear kinetics of drugs administered intra-tympanically as solutions show rapid decline in concentration within the middle ear cavity, e.g., dexamethasone phosphate, which fell to 10% of the applied concentration at 93 min after injection into the middle ear (Salt and Plontke, 2018). This process could be delayed by the use of slow-release vehicles. The delayed elimination (Hellberg et al., 2013) and prolonged retention of cisplatin for months to years in the cochlea creates challenges for intratympanic protection strategies (Breglio et al., 2017).

### Cisplatin Ototoxicity

Cisplatin is frequently used to treat a variety of malignant solid tumors. These include ovarian and testicular cancer, head and neck carcinomas, cervical, bladder, and lung cancer. Although cisplatin is a quite effective antineoplastic drug, dose limiting side effects often occur. Such unintended toxicities include: ototoxicity, neurotoxicity, nephrotoxicity, and bone marrow toxicity. Investigation of potential protective agents to ameliorate cisplatin ototoxicity have not yet yielded an effective drug that has been approved by the United States Food and Drug Administration (FDA). Of great concern is the potential interference of cisplatin efficacy when systemic otoprotective drugs are administered. These otoprotective drugs could neutralize or diminish the anti-tumor effects of cisplatin and may also produce additional side effects, including communication, learning, cognition, and quality of life (Brooks and Knight, 2018). Therefore, numerous preclinical studies have investigated potential protective agents against cisplatin ototoxicity using local therapy, such as intra-tympanic injection. Cisplatin-induced hearing loss is bilateral and permanent. The hearing loss occurs mostly in the high frequencies. It can drastically compromise the quality of life for cancer survivors. Therefore the use of intra-tympanic therapy is attractive since the concentration of putative protective agents will be greater in the basal turn of the cochlea where high frequency hearing is transduced.

### Mechanisms Underlying Cisplatin Ototoxicity

Cisplatin ototoxicity and the underlying mechanisms are still being investigated. Several mechanisms have been proposed. These include oxidative stress caused by the production of reactive oxygen species (Kros and Steyger), which can be mediated by activation of a cochlear specific isoform of the enzyme NADPH oxidase (NOX3), and by up regulation of transient receptor potential vanilloid 1 (TRPV1) channels (Mukherjea et al., 2008, 2011; Sheth et al., 2017). Cisplatin mediated damage to mitochondria resulting in cleavage of caspases leading to apoptosis of critical structures in the cochlea (outer hair cells, cells of the stria vascularis and spiral ligament, and spiral ganglion cells); DNA damage with activation of p53 leading to activation of activation of signal transducer and activator of transcription 1 (STAT1) (Zhang et al., 2003; Kaur et al., 2011; Benkafadar et al., 2017; Bhatta et al., 2019), resulting in inflammation and apoptosis (Sheth et al., 2017). Recent reviews of the proposed mechanisms of cisplatin ototoxicity have been published (Karasawa and Steyger, 2015, Sheth et al., 2017, Kros and Steyger, 2018).

### Intra-Tympanic Treatments for Cisplatin Ototoxicity

A wide variety of putative protective agents have been reported to ameliorate cisplatin ototoxicity when administered by intra-tympanic injection. A short list of some successful otoprotective agents administered intra-tympanically prior to cisplatin treatment *in vivo* have been categorized as inhibitors, biologicals, siRNA, and dexamethasone and have been listed below:

• Kenpaullone is an inhibitor of multiple kinases, including cyclin-dependent kinase 2 (CDK2). Significant otoprotection was demonstrated in both mice and rats. Mice receiving intra-tympanic kenpaullone demonstrated significant reductions of ABR threshold elevation, at frequencies of 16 and 32 kHz. Morphology of OHCs in the 32 kHz region showed significant protection in kenpaullone treated mice. Even more robust findings were demonstrated in rats. Intra-tympanic kenpaullone provided complete protection against cisplatin ototoxicity in the rat. These findings support the hypothesis that CDK2 inhibition by kenpaullone ameliorates cisplatin ototoxicity by inhibiting mitochondrial ROS production and also preventing cochlear cell death mediated by caspase-3/7 (Teitz et al., 2018).
• Copper sulfate, a copper transporter-1 inhibitor, when administered intra-tympanically 30 min prior to intraperitoneal cisplatin in mice showed significant protection against threshold shifts in ABR using click stimuli and pure tones at 8, 16, and 32 kHz. However, concerns were expressed about the toxicity of copper sulfate. This led to the suggestion that other less toxic inhibitors of CTR1 should be developed and tested (More et al., 2010).
• Thiosulfate, an antioxidant was administered as thiosulphate-hyaluronan gel into the tympanic cavity of guinea pigs 3 h prior to intravenous cisplatin injection. This resulted in high concentrations of thiosulfate in the perilymph of scala tympani and it protected against cochlear hair cell loss from cisplatin. Levels of thiosulfate in blood were kept low, avoiding potential chelation of cisplatin in the blood that could interfere with the anti-tumor efficacy of cisplatin (Berglin et al., 2011).
• KR-22332 (3-amino-3-(4-fluoro-phenyl)-1H-quinoline-2, 4-dione) is a novel compound that suppresses ROS. Intra-tympanic administration of KR-22332 in rats protected against cisplatin induced ABR threshold shift to click stimuli. This compound inhibited cisplatin-induced up regulation of NOX3 in the cochlea and reduced the activation of p53, MAP kinases, caspase 3 and tumor necrosis factor-α (TNF-alpha), and TUNEL expression in rat cochlea. KR-22332 may ameliorate cisplatin ototoxicity by reducing the generation of ROS and by preventing mitochondrial dysfunction (Shin et al., 2013).
• Antioxidant vitamins such as vitamin E and vitamin C have been tested for protection against cisplatin ototoxicity. Trolox, a water-soluble form of alpha-tocopherol is an antioxidant. It was applied locally on the round window of guinea pigs treated with cisplatin. Trolox administered in combination with cisplatin prevented ABR threshold elevations and protected against the loss of hair cells (Teranishi and Nakashima, 2003). Another study in rats looked at the effect of intra-tympanic application of vitamin E solution followed by cisplatin administration 30 min later. Significant protection against cisplatin induced ABR threshold shifts was seen in rats (Paksoy et al., 2011). Another strategy employed the intra-tympanic administration of vitamin E polymeric nanoparticles in rats treated with cisplatin. Rats pretreated with vitamin E nanoparticles had significant protection against cisplatin-induced ABR threshold shifts at 12, 20, and 32 (Martin-Saldana et al., 2017). Vitamin C administered by intra-tympanic injection protected against cisplatin-induced decrease in DPOAE amplitudes in rats treated with cisplatin (Celebi et al., 2013).
• Melatonin is a hormone secreted by the pineal gland that has antioxidant properties. It has both indirect antioxidant and direct free radical scavenger activity. Rats treated with intra-tympanic melatonin showed improved ABR thresholds for clicks, 4, 6, and 8 kHz and threshold shifts for DPOAE. Staining for TNF-alpha was diminished in melatonin treated rats receiving cisplatin (Demir et al., 2015).
• Capsaicin is a spicy capsaicinoid, a natural product produced by hot chili peppers, Capsicum fruits. This alkaloid has been used for its analgesic and anti-inflammatory actions (Lavorgna et al., 2019), Capsaicin activates TRPV1 pain receptors, and can produce rapid desensitization of TRPV1. The intra-tympanic administration of capsaicin in rats 24 h prior to cisplatin reduced ABR threshold shifts. Capsaicin appears to prevent cisplatin ototoxicity by increasing the expression of cannabinoid 2 receptors (CB2R) in the cochlea leading to increased activation of pro-survival transcription factor signal transducer and activator of transcription (STAT3) (Bhatta et al., 2019).
• JWH-015 (2-methyl-1-propyl-1H-indol-3-yl)-1-naphthalenylmethanone) is a cannabinoid receptor 2 (CB2) agonist. Pretreatment with intra-tympanic JWH-015, 30 min prior to cisplatin reduced ABR threshold shifts at 8, 16, and 32 kHz and also protected against the loss of OHCs in rats. In addition, this CB2R agonist prevented cisplatin-induced loss of ribbon synapses on inner hair cells (IHCs) and prevented loss of Na^+^/K^+^-ATPase immunoreactivity in the stria vascularis (Ghosh et al., 2018).
• Pifithrin-alpha is an inhibitor of p53. Pifithrin-alpha was applied on the RWM of the chinchilla prior to the local application of cisplatin. The cochleae that were pretreated with pifithrin were significantly protected from cisplatin-induced increase in ABR threshold shifts at 1,2,4,8, and 16 kHz (Parhizkar and Rybak, 2003).
• R-PIA (R-phenylisopropyladenosine) is an adenosine A1 receptor agonist. Intra-tympanic administration of R-PIA in rats prior to cisplatin reduced cisplatin-induced ABR threshold elevation and OHCs were preserved. This protection was associated with reduced NOX3 expression, STAT1 activation, TNF-α levels, and apoptosis in the cochlea (Kaur et al., 2016).
• D-methionine and L-methionine are amino acids with antioxidant properties and both of these compounds have been shown to protect against cisplatin ototoxicity in preclinical studies. D-methionine applied to the RWM provided complete protection against cisplatin applied to the round window in chinchillas. ABR thresholds and OHCs were completely preserved in animals pretreated with D-methionine (Korver et al., 2002). In a study using guinea pigs, osmotic pumps were implanted to provide continuous administration of D-methionine, sodium thiosulfate, fibroblast growth factor-2, or brain-derived neurotrophic factor in animals treated with cisplatin. Guinea pigs receiving D-methionine demonstrated better OAEs on the 3th and 5th day of a 5 day regimen of cisplatin administration. On 5th and 6th day of the treatment, D-methionine failed to provide protection. It appears that the additional dosing of cisplatin overpowered the effectiveness of D-methionine on those later 2 days. The other agents provided no significant protection (Wimmer et al., 2004). The efficacy of L-methionine against cisplatin ototoxicity was investigated in rats. Local application of L-methionine prior to cisplatin provided complete protection against cisplatin-induced ABR threshold shifts and preserved the integrity of OHCs against damage by cisplatin (Li et al., 2001).
• L-N-acetylcysteine (L-NAC) is a sulfhydryl compound that can neutralize cisplatin and function as an antioxidant. A 2% solution of L-NAC was administered by intra-tympanic injection in guinea pigs treated with cisplatin. Pretreatment with L-NAC preserved DPOAEs that were otherwise severely affected by cisplatin. This same study successfully utilized lactated Ringer’s solution by intra-tympanic injection prior to cisplatin administration. The latter solution was also effective in preserving DPOAEs in cisplatin treated guinea pigs (Choe et al., 2004). A later study showed that intra-tympanic administration of L-NAC was harmful and exacerbated cisplatin ototoxicity in the guinea pig. However, this latter study utilized extremely high concentrations of L-NAC (20%) and this proved to be toxic. Animals receiving this high dose of L-NAC showed severe disruption of OHC stereocilia (Nader et al., 2010). It appears that a more dilute solution of L-NAC is a better preparation to use for intra-tympanic injection to ameliorate cisplatin ototoxicity.
• TNF-alpha antagonist, etanercept, when administered intra-tympanically in rats protected against OHC damage and cisplatin-induced hearing loss. ABR threshold shifts were significantly reduced in rats treated with etanercept 30 min prior to cisplatin. Scanning electron microscopy of etanercept pre-treated animals showed significant protection against cisplatin induced OHC damage (Kaur et al., 2011).
• RNA silencing has been successfully employed using intra-tympanic delivery for protection against cisplatin ototoxicity. In a rat model of cisplatin ototoxicity, it was shown than intra-tympanic administration of siRNA to knock down TRPV1 protected against cisplatin-induced hearing loss and damage to outer hair cells in the cochlea (Mukherjea et al., 2008). It was hypothesized that protection was afforded by reducing down-stream targets, such as the cochlear specific NADPH oxidase -3 (NOX3) enzyme, and STAT-1. Indeed, the intra-tympanic injection of siRNA directed against NOX3 (Mukherjea et al., 2011) or STAT-1 siRNA (Kaur et al., 2011) were each protective against cisplatin induced hearing loss and outer hair cell damage. NOX3 activation results in reactive oxygen species upregulation and STAT-1 can promote inflammation and apoptosis in the cochlea as a result of cisplatin’s ototoxic effect. Such deleterious effects can be prevented by the use of these siRNAs.
• Dexamethasone is a glucocorticoid that appears to offer protection against cisplatin ototoxicity by several mechanisms. These include the down-regulation of pro-inflammatory genes that regulate the expression of cytokines; the inhibition of apoptosis; the up-regulation of antioxidant enzymes that could antagonize the effects of ROS (Hazlitt et al., 2018).
  - Successful attenuation of cisplatin ototoxicity has been reported in various animal models treated with intra-tympanic dexamethasone. The animals tested include: mouse (Hill et al., 2008), aged mouse (Parham, 2011), rat (Paksoy et al., 2011), and guinea pig (Shafik et al., 2013). Intratympanic dexamethasone delivered 1 day before cisplatin treatment did not protect against cisplatin ototoxicity. However, intra-tympanic dexamethasone administered 1 h before cisplatin provided significant preservation of cochlear structure and function (Shafik et al., 2013). The efficacy of intra-tympanic dexamethasone solution in protecting against cisplatin ototoxicity in various experimental animal models has been rather inconsistent. Results appear to depend on the dose of both dexamethasone and cisplatin and the species of experimental animals (Hazlitt et al., 2018). Therefore, other formulations have been explored for providing sustained release of steroid or increase in penetration into the cochlea, such as the incorporation of dexamethasone into nanoparticles.
  - Dexamethasone OTO-104 contains micronized dexamethasone in a poloxamer based hydrogel. This formulation was found to be much more effective than dexamethasone solution alone (Fernandez et al., 2016). A single intra-tympanic injection of 6% OTO-104, provided nearly total protection against cisplatin ototoxicity in guinea pigs receiving acute injection of cisplatin. On the other hand intra-tympanic dexamethasone solution offered no protection. OTO-104 was also very effective in prevention of hearing loss associated with chronic administration of cisplatin (Fernandez et al., 2016).
  - Dexamethasone has also been delivered intra-tympanically as nanoparticles. Dexamethasone-PEG-PLA nanoparticles provided significant otoprotection against cisplatin induced ABR threshold shifts at 4 and 8 kHz but not at 16 or 24 kHz in guinea pigs (Sun et al., 2015). Dexamethasone polymeric nanoparticles also protected against cisplatin ototoxicity in rats (Martin-Saldana et al., 2017). Dexamethasone treatment by bullostomy (intra-tympanic administration) successfully reduced hearing loss in all frequencies (from 8 to 32 kHz) tested by auditory steady-state responses (ASSR) (Martin-Saldana et al., 2017).
  - Dexamethasone-A666 nanoparticles administered intra-tympanically protected guinea pigs against cisplatin-induced cochlear outer hair cell damage and hearing loss (Wang et al., 2018). This latter study used A666-peptides that were shown to bind to prestin in outer hair cells. This formulation effectively delivered dexamethasone into outer hair cells and was significantly more effective than intra-tympanic injection of free dexamethasone or dexamethasone incorporated into nanoparticles without A666 (Wang et al., 2018).
• Prednisolone was found to reduce cisplatin induced ABR threshold elevations in mice. Intra-tympanic magnetically delivered prednisolone-loaded nanoparticles resulted in significantly lower elevations of ABR threshold, particularly at the higher frequencies (16 and 32 kHz) compared with intra-tympanic methylprednisolone solution or empty magnetic nanoparticles (Ramaswamy et al., 2017).

We have summarized the studies reporting intra-tympanic drug delivery that protect against cisplatin ototoxicity in Table 1.

**TABLE 1:** This table summarizes pertinent studies demonstrating amelioration of cisplatin-induced ototoxicity using intra-tympanic therapy.

**Drug**	**Animal model**	**Mechanism**	**References**
Kenpaullone	Mouse, Rat	Cyclin-dependent kinase-2 inhibitor	Teitz et al.,2018
Etanercept	Rat	TNF-alpha inhibitor	Kaur et al.,2011
Copper sulfate	Mouse	CTR1 inhibitor	More et al.,2010
Thiosulfate-hyaluronan gel	Guinea pig	Platinum chelator	Berglin et al.,2011
KR-22332 (3-amino-3-(4-fluoro-phenyl)-1H-quinoline- 2,4-dione)	Rat	Suppresses ROS	Shin et al.,2013
Trolox	Guinea pig	Antioxidant	Teranishi and Nakashima,2003
Vitamin E	Rat	Antioxidant	Paksoy et al.,2011
Vitamin E polymeric nanoparticles	Rat		Martin-Saldana et al.,2017
Vitamin C	Rat	Antioxidant	Celebi et al.,2013
Melatonin	Rat	Antioxidant	Demir et al.,2015
Capsaicin	Rat	CB2R upregulation increase STAT3/STAT1	Bhatta et al.,2019
Dexamethasone	Rat	Anti-inflammatory	Paksoy et al., 2011; Özel et al.,2016
	Mouse		Hill et al.,2008
	Aged mouse		Parham,2011
	Guinea pig		Murphy and Daniel, 2011; Shafik et al.,2013
Dexamethasone-PEG-PLA nanoparticles	Guinea pig		Sun et al.,2015
Dexamethasone polymeric nanoparticles	Rat		Martin-Saldana et al.,2017
Dexamethasone-A666 nanoparticles	Guinea pig		Wang et al.,2018
Dexamethasone OTO-104	Guinea pig	Antioxidant	Fernandez et al.,2016
Prednisolone magnetic nanoparticles	Mouse	Anti-inflammatory	Ramaswamy et al.,2017
JWH-015	Rat	CB2R upregulation	Ghosh et al.,2018
Pifithrin-alpha	Chinchilla	p53 inhibitor	Parhizkar and Rybak,2003
R-PIA	Rat	Adenosine A1R	Kaur et al.,2016
D-methionine	Chinchilla	Antioxidant	Korver et al.,2002
	Guinea pig		Wimmer et al.,2004
L-methionine	Rat	Antioxidant	Li et al.,2001
L-N-acetylcysteine	Guinea pig	Antioxidant	Choe et al.,2004
Lactated Ringer’s	Guinea pig	–	Choe et al.,2004
TRPV1 siRNA	Rat	Decrease ROS	Mukherjea et al.,2008
NOX3 siRNA	Rat	Decrease ROS	Mukherjea et al.,2011
STAT1 siRNA	Rat	Anti-inflammatory	Kaur et al.,2011

### Noise Induced Hearing Loss and Underlying Mechanisms

Noise induced hearing loss (NIHL) is a global burden with an estimated 16% of the adult population being affected, with significant regional variations (Nelson et al., 2005; Oishi and Schacht, 2011; Basner et al., 2014; Masterson et al., 2018). NIHL is not only characterized by increased thresholds in hearing, speech processing and tinnitus but also associated with sleep disorders, cardiovascular diseases and cognitive decline (Gates et al., 2000; Ohlemiller, 2008; Kumar et al., 2012; Bressler et al., 2017; Cunningham and Tucci, 2017; Le Prell and Clavier, 2017; Munzel et al., 2018).

The sensitivity to noise varies with the intensity and duration of exposure and the mammalian species tested. Auditory threshold shifts after noise exposure can cause either a temporary threshold shift (TTS) or shifts that do not revert back to baseline are known as permanent threshold shifts (PTS) (Kujawa and Liberman, 2009). Permanent hearing loss or PTS occurs when the noise exposure exceeds the capacity of the cochlea to recover. Permanent damage can be inflicted upon various cochlear tissues, including hair cells, spiral ganglion neurons and the lateral wall (stria vascularis and spiral ligament). Intense noise can cause mechanical damage that can result in the mixing of endolymph and perilymph causing high levels of potassium to kill hair cells (Kurabi et al., 2017). NIHL could be caused by a number of molecular events in the cochlea. Acoustic trauma can lead to the production of reactive oxygen species (Kros and Steyger) in the cochlea. ROS can remain in the cochlea for up to 10 days after noise exposure (Yamane et al., 1995). ROS may be generated by enzymes activated by noise exposure, including NADPH oxidases. ROS can oxidize lipids to form vasoactive lipid peroxidation molecules like isoprostanes (Ohinata et al., 2000). These toxic products may reduce cochlear blood flow. ROS can also lead to the formation of inflammatory cytokines that can cause cochlear damage. These include interleukin-6 and tumor necrosis factor-alpha (Kurabi et al., 2017). Reactive nitrogen species (RNS) are also formed in the cochlea of animals subjected to high levels of noise. These products include nitro tyrosine and peroxynitrite (Ohinata et al., 2000). The latter toxic free radical is formed by the reaction of nitric oxide (NO) with superoxide. Toxic noise exposure can produce accumulation of calcium in the inner ear tissues. Excess calcium may cause ROS release from mitochondria and can upregulate mitogen activated kinase (MAPK) including c-Jun-N-terminal kinase (JNK) and other cellular stress molecules. These downstream molecules can lead to OHC apoptosis or necrosis (Kurabi et al., 2017).

### Intra-Tympanic Treatments Against Noise Trauma

Protective agents have been reported to ameliorate NIHL when administered by intra-tympanic injection. The following is a short list of some successful otoprotective agents administered intra-tympanically *in vivo*. Interestingly, some of these inhibitors were also protective against cisplatin induced hearing loss. Most otoprotective agents used are either inhibitors of cellular pathways, antioxidants, anti-inflammatory compounds, siRNA trophic factors or dexamethasone and have been listed below:

• A cell-permeable inhibitor of JNK mediated apoptosis, AM-111, was administered on the RWM (in a hyaluronic acid gel formulation or osmotic mini-pump) 1 or 4 h after impulse noise exposure in chinchillas. Three weeks after traumatic noise exposure the PTS were significantly less in animals receiving AM-111 even when it was administered 4 h after noise exposure (Coleman et al., 2007). D-JNKI-1 was found to block the mitogen-activated protein kinase/JNK-mediated activation of a mitochondrial death pathway. D-JNKi-1 administered intra-tympanically to guinea pigs exposed to acoustic trauma also provided excellent protection. The majority of hair cells were preserved in the area of maximum noise damage and resulted in almost no permanent hearing loss. Treatment was effective even when administered up to 12 h after noise exposure. These findings strongly suggest that the mitogen-activated protein kinase/JNK signaling pathway plays a critical role in producing hair cell death from acoustic trauma (Wang et al., 2007).
  - A novel and intriguing refinement of the intra-tympanic delivery of D-JNKi-1 to the cochlea of mice was recently reported. Mice underwent intra-tympanic application of a chitosan glycerophosphate (CGP)-hydrogel system containing targeted and untargeted D-JNKi-1 containing multifunctional nanoparticles (MFNPs) or empty MFNPs. Targeting was directed to the protein prestin in OHCs. Two days after round window application of the hydrogel the mice were exposed to acoustic trauma. ABR threshold shifts at 14 days after noise exposure were significantly lower for 4 and 8 kHz stimuli in mice treated with targeted MFNPs containing D-JNKi-1 compared to untargeted D-JNKi-l MFNPs but protection was similar at 16, 24 and 32 kHz. At these frequencies, both targeted and untargeted D-JNKi-l-MFNPs provided partial protection that did not significantly differ from each other (Kayyali et al., 2018).
• Rosmarinic acid is a polyphenol that is found in aqueous extracts of spearmint. It has demonstrated antioxidant, anti-inflammatory and neuroprotective properties (Falcone et al., 2019). Rats were exposed to acoustic trauma and underwent ABR measurements before and up to 30 days afterward. One group was treated with systemic rosmarinic acid and a second treatment group was administered intra-tympanic rosmarinic acid. Significant protection against ABR threshold shifts was seen in both treatment groups compared with controls. Less OHC loss and decreased evidence of superoxide production and lipid peroxidation was ascertained using dihydroethidium and 8-isoprostane immunostaining, respectively. These findings strongly suggest that adminstration of rosmarinic acid by both routes of administration protected the hearing and preserved the cochlea of rats exposed to noise trauma (Fetoni et al., 2018).
• Peroxisome proliferator-activated receptors (PPARs) function as lipid sensors and help to regulate redox balance by inhibiting ROS and upregulating antioxidant genes. Pioglitazone is a PPAR-gamma agonist that has been shown to reduce inflammation in patients with type two diabetes and coronary artery disease. This drug seemed to have favorable properties to test as a protective agent against noise trauma. Rats were administered pioglitazone in a temperature sensitive gel intra-tympanically 1 h after acoustic trauma. Pioglitazone significantly protected against threshold shifts in the ABR and significantly reduced the loss of OHCs. These findings were associated with a reduction in superoxide anion expression and lipid peroxidation (8-isoprostane). Anti-inflammatory effects of pioglitazone were demonstrated by its blockade of noise induced upregulation of pNFkB and interleukin 1b (IL-1b). Thus, pioglitazone protection against traumatic noise injury to the cochlea by both anti-oxidant and anti-inflammatory effects (Paciello et al., 2018).
• Caroverine is an antagonist of two glutamate receptors, N-methyl-D-aspartate (NMDA) and alpha-amino-3-hydroxy-5-methyl-4-isoxazolepropionic acid (AMPA). Caroverine was applied onto the RWM with gelfoam in guinea pigs, followed by noise exposure. ABR threshold shifts were significantly lower in caroverine treated animals (Chen et al., 2004).
• Edaravone is a free radical scavenger and antioxidant. Edaravone solid lipid nanoparticles, were delivered to guinea pigs by intra-tympanic injection. Noise exposure resulted in ABR threshold shifts and induced ROS formation. Edaravone reduced the ABR threshold shift and ROS production in noise-exposed animals compared with controls. Edaravone solid lipid nanoparticles show protective effects against noise-induced hearing loss. However, guinea pigs treated with edaravone had no significant protection of OHCs. More experiments will be needed to see if edaravone could be useful in protecting cochlear tissues from noise injury (Gao et al., 2015).
• Kenpaullone is an inhibitor of CDK2. When kenpaullone was injected intra-tympanically in mice had significantly better ABR thresholds and wave 1 amplitudes than controls. In animals treated with this agent, the presynaptic ribbon density at D14 after the acoustic damage was diminished. These data support the hypothesis that kenpaullone protects against noise-induced hearing loss in mice. It is interesting to note that kenpaullone also protected against cisplatin (see above) (Teitz et al., 2018).
• RNA silencing: Noise exposed mice suffered permanent ABR threshold shifts, loss of OHCs and cochlear synapses. G9a (KMT1C, EHMT2) is an important histone lysine methyltransferase encoded by the human *EHMT2* gene and responsible for histone H3 lysine 9 dimethylation (H3K9me2). The intra-tympanic administration of siRNA against G9a to silence the *EHMT2* gene 72 h prior to noise exposure significantly reduced ABR threshold shifts and resulted in greater survival of OHCs compared to treatment with the control siRNA. These data suggest that pretreatment with siG9a partially ameliorates noise-induced permanent hearing loss via the inhibition of G9a (Xiong et al., 2019).
  - Noise exposure activates two key enzymes in the cochlea of mice: phosphorylated AMP-activated protein kinase-alpha-1 (AMPK-alpha-1) and its upstream kinase, liver kinase B1 (LKB1) in the cochlea. Pretreatment with intra-tympanic siRNA against AMPK-alpha-1 prior to noise exposure inhibited the expression of this enzyme and significantly reduced ABR threshold shifts and loss of OHCs and loss of synaptic ribbons at IHCs. Furthermore, inhibition of LKB1 by intra-tympanic siRNA reduced the noise-induced increase in phosphorylation of AMPK-alpha-1 in OHCs, reduced the loss of IHC synaptic ribbons and OHCs, and protected against ABR threshold shifts. These findings provide interesting new approach to prevent noise-induced hearing loss and cochlear synaptopathy (Hill et al., 2016).
• Neurotrophins have been used successfully for preservation of IHC pre and post-synaptic ribbon synapses: Guinea pigs were exposed for 2 h to 4 to 8 kHz noise at 95 dB. Auditory brainstem responses to pure-tone pips were acquired preoperatively, and at 1 and 2 weeks’ post exposure. Immediately after noise exposure neurotrophins (brain-derived neurotrophic factor and neurotrophin-3) were applied to the RWM. ABR amplitude growth recovered in the ears of neurotrophin-treated guinea pigs using 16 kHz tones. Significantly more presynaptic ribbons, post-synaptic glutamate receptors, and co-localized ribbon synapse were seen after neurotrophin treatment. These findings supported the hypothesis that the local application of neurotrophins to the round window immediately after noise exposure will prevent noise-induced “hidden hearing loss” (Sly et al., 2016).
  - Even more exciting is the report that synapses may regenerate with intra-tympanic treatment with NT-3 after noise exposure. Mice exposed to noise (“neuropathic noise”) that resulted in loss of up to 50% of synapses in the base of the cochlea within 24 h were treated with intra-tympanic neurotrophic-3 (NT-3 in a poloxamer gel) 24 h after noise exposure. Interestingly, this treatment was associated with regeneration of both pre- and post-synaptic elements at the junction of the IHC and cochlear nerve. Not only did the mice show structural recovery of these synapses, but they also demonstrated functional recovery by restoration of ABR wave 1 suprathreshold amplitudes. These findings have significant potential for healing “hidden hearing loss” in humans (Suzuki et al., 2016).
• Dexamethasone is the most frequently tested glucocorticosteroid by intra-tympanic injection to protect against noise-induced hearing loss. Rats were exposed to noise at 110 dB for 25 min and DPOAE measurements were performed before and after noise exposure. DPOAE measurements were performed before and 7 and 10 days after noise trauma. Rats treated with intra-tympanic dexamethasone had significantly better hearing than controls (Gumrukcu et al., 2018). Guinea pigs receiving intra-tympanic dexamethasone demonstrated significantly smaller ABR threshold shifts and decreased OHC loss compared with controls. They also had significant reduction in malondialdehyde concentration in the cochlea. This suggests that dexamethasone provided antioxidant effects in the treated ears (Chi et al., 2011). Another study showed short-term protection against hearing loss in guinea pigs with intra-tympanic dexamethasone. Animals received dexamethasone by intra-tympanic injection 2 h prior to white noise exposure. ABR thresholds were better and hair cell loss was reduced by this treatment. However, the major flaw of this study is that ABR thresholds and cochlear histology were performed only 2 h after noise exposure (Heinrich et al., 2016). Another study using guinea pigs tested the efficacy of dexamethasone administered intra-tympanically 2 h prior to white noise exposure. One group received dexamethasone solution and a second group was provided dexamethasone microbubbles with ultrasound irradiation. Compared with controls, dexamethasone in both groups provided protection against hair cell loss and auditory threshold shifts. However, significantly greater protection was afforded to the guinea pigs pretreated with ultrasound delivered microbubbles (Shih et al., 2018).

• Interesting findings were reported in a study of noise exposed mice. Animals were exposed to 110 db white noise for 60 min in a single exposure. One group received intraperitoneal dexamethasone injection (IP) daily for five consecutive days, while another cohort was given intra-tympanic injection of dexamethasone on days one and four after noise exposure. Mice in both treatment groups showed improved ABR thresholds but no apparent improvement in DPOAEs. Interestingly, better preservation of organ of Corti ultrastructure was observed in mice receiving IP drug than in those who were administered dexamethasone by intra-tympanic injection. On the other hand, efferent synapses were damaged in control (noise only), and in both groups treated with dexamethasone. However, there was better preservation of synapses of efferent terminals on OHCs in the group treated with intra-tympanic steroids (Han et al., 2015).
• In efforts to provide sustained release of dexamethasone for prolonged otoprotection against noise the efficacy of OTO-104 was investigated both prior to and following acute acoustic trauma. OTO-104 is a poloxamer-based hydrogel containing micronized dexamethasone. Guinea pigs received a single intra-tympanic injection of OTO-104 and were assessed in a model of acute acoustic trauma. Doses of at least 2.0% OTO-104 offered significant protection against hearing loss induced by noise exposure when administered 1 day prior to trauma and up to 3 days afterward. Otoprotection remained effective even with higher degrees of trauma. In contrast, the administration of a dexamethasone sodium phosphate solution did not protect against noise-induced hearing loss. Activation of the classical nuclear glucocorticoid and mineralocorticoid receptor pathways was required for otoprotection by OTO-104. The sustained release features of OTO-104 provided greater protection than the solution (Harrop-Jones et al., 2016).
• Methylprednisolone was administered intra-tympanically to guinea pigs exposed to impulse noise. Animals receiving this treatment had significantly better ABR thresholds at 4 weeks compared with those treated with saline. Significantly better preservation of hair cells was observed in the cochleae of guinea pigs receiving intra-tympanic methylprednisolone compared to those treated with saline (Zhou et al., 2009). Intra-tympanic methylprednisolone injection in rats administered following acoustic trauma was shown to reduce OHC loss. Although DPOAE measurement within the first week demonstrated significantly better amplitudes in the treated rats compared to controls at 2 weeks, there was no significant difference in DPOAE amplitudes between the treated and control group (Ozdogan et al., 2012).

We have summarized the studies reporting intra-tympanic drug delivery that protect against NIHL in Table 2.

**TABLE 2:** This table summarizes pertinent studies of amelioration of noise-induced hearing loss using intra-tympanic therapy.

**Drug**	**Animal model**	**Mechanism**	**References**
AM-111 (D-JNKi-1)	Chinchilla	Anti-apoptotic	Coleman et al.,2007
d-JNKI-1	Guinea pig	Anti-apoptotic	Wang et al.,2007
D-JNKi-1 multifunctional	Mouse	Anti-apoptotic	Kayyali et al.,2018
Methylprednisolone	Guinea pig, Rat	Anti-inflammatory	Zhou et al., 2009; Ozdogan et al.,2012
Dexamethasone	Guinea pig	Anti-inflammatory	Chi et al., 2011; Heinrich et al.,2016
	Mouse		Han et al.,2015
	Rat		Gumrukcu et al.,2018
Dexamethasone (OTO-104)	Guinea pig		Harrop-Jones et al.,2016
Dexamethasone ultrasonic microbubbles	Guinea pig		Shih et al.,2018
Caroverine	Guinea pig	Glutamate antagonism	Chen et al.,2004
Kenpaullone	Mouse	Cyclin-dependent kinase-2 inhibitor	Teitz et al.,2018
Edavarone solid lipid nanoparticles	guinea pig	Antioxidant	Gao et al.,2015
BDNF + NT3	Guinea pig	Synapse regeneration	Sly et al.,2016
NT3	Mouse	Synapse regeneration	Suzuki et al.,2016
Pioglitazone	Rat	Anti-inflammatory, Antioxidant	Paciello et al.,2018
Rosmarinic acid	Rat	Antioxidant	Fetoni et al.,2018
AMPK-alpha1 siRNA	Mouse	*AMPK-alpha 1*	Hill et al.,2016
LKB1 siRNA	Mouse	*LKB1*	Hill et al.,2016
G9a siRNA	Mouse	*EHMT2*	Xiong et al.,2019

## Conclusion

Studies described in the above review highlight some exciting new research on local drug delivery using intra-tympanic administration of substances to ameliorate ototoxicity of cisplatin and noise-induced hearing loss. These are two very important causes of permanent sensorineural hearing loss for which there are currently no approved treatments on the market. The reports that are discussed include the proposed mechanisms for protection against these two major causes of hearing loss in humans.

The advantages of local delivery include targeted effects on the inner ear while minimizing systemic toxicity or interference with cisplatin antitumor efficacy and the ability to deliver sufficient amount of protective agent within the inner ear while by-passing the blood-labyrinth barrier (BLB), a major obstacle to effective protection delivered by systemic administration. The use of intra-tympanic injection in humans is minimally invasive and can generally be performed in the office under local anesthesia. The exploration of methods to extend the duration of release of protective agents and the investigation of round window permeation enhancers can provide higher concentrations of protectant molecules in the cochlea following intra-tympanic administration. The use of nanoparticles incorporating protective agents to target prestin in outer hair cells is very innovative and exciting. Although the regeneration of hair cells in the cochlea of humans has not been demonstrated, the regeneration of synapses on IHCs in animals after noise-induced synaptopathy using locally applied neurotrophins appears feasible. Future research is likely to reveal new mechanisms and exciting and novel treatments for sensorineural hearing loss.

## Author Contributions

LR conceived the study. LR and AD wrote the manuscript. DM and VR critiqued and revised the manuscript.

## Conflict of Interest Statement

The authors declare that the research was conducted in the absence of any commercial or financial relationships that could be construed as a potential conflict of interest.

## References

[B1] Basner M. Babisch W. Davis A. Brink M. Clark C. Janssen S. (2014). Auditory and non-auditory effects of noise on health. *Lancet* 383 1325–1332. 10.1016/s0140-6736(13)61613-x 24183105 PMC3988259

[B2] Benkafadar N. Menardo J. Bourien J. Nouvian R. François F. Decaudin D. (2017). Reversible p53 inhibition prevents cisplatin ototoxicity without blocking chemotherapeutic efficacy. *EMBO Mol. Med.* 9 7–26. 10.15252/emmm.201606230 27794029 PMC5210089

[B3] Berglin C. E. Pierre P. V. Bramer T. Edsman K. Ehrsson H. Eksborg S. (2011). Prevention of cisplatin-induced hearing loss by administration of a thiosulfate-containing gel to the middle ear in a guinea pig model. *Cancer Chemother. Pharmacol.* 68 1547–1556. 10.1007/s00280-011-1656-2 21533919

[B4] Bhatta P. Dhukhwa A. Sheehan K. Al Aameri R. F. H. Borse V. Ghosh S. (2019). Capsaicin protects against cisplatin ototoxicity by changing the STAT3/STAT1 ratio and activating cannabinoid (CB2) receptors in the cochlea. *Sci. Rep.* 9:4131. 10.1038/s41598-019-40425-9 30858408 PMC6411993

[B5] Breglio A. M. Rusheen A. E. Shide E. D. Fernandez K. A. Spielbauer K. K. McLachlin K. M. (2017). Cisplatin is retained in the cochlea indefinitely following chemotherapy. *Nat. Commun.* 8:1654. 10.1038/s41467-017-01837-1 29162831 PMC5698400

[B6] Bressler S. Goldberg H. Shinn-Cunningham B. (2017). Sensory coding and cognitive processing of sound in Veterans with blast exposure. *Hear. Res.* 349 98–110. 10.1016/j.heares.2016.10.018 27815131 PMC5645017

[B7] Brooks B. Knight K. (2018). Ototoxicity monitoring in children treated with platinum chemotherapy. *Int. J. Audiol.* 57 S34–S40. 10.1080/14992027.2017.1355570 28737048

[B8] Celebi S. Gurdal M. M. Ozkul M. H. Yasar H. Balikci H. H. (2013). The effect of intratympanic vitamin C administration on cisplatin-induced ototoxicity. *Eur. Arch. Otorhinolaryngol.* 270 1293–1297. 10.1007/s00405-012-2140-2 22907028

[B9] Chen Z. Ulfendahl M. Ruan R. Tan L. Duan M. (2004). Protection of auditory function against noise trauma with local caroverine administration in guinea pigs. *Hear. Res.* 197 131–136. 10.1016/j.heares.2004.03.021 15504611

[B10] Chi F. L. Yang M. Q. Zhou Y. D. Wang B. (2011). Therapeutic efficacy of topical application of dexamethasone to the round window niche after acoustic trauma caused by intensive impulse noise in guinea pigs. *J. Laryngol. Otol.* 125 673–685. 10.1017/S0022215111000028 21693072

[B11] Choe W. T. Chinosornvatana N. Chang K. W. (2004). Prevention of cisplatin ototoxicity using transtympanic N-acetylcysteine and lactate. *Otol. Neurotol.* 25 910–915. 10.1097/00129492-200411000-00009 15547419

[B12] Coleman J. K. Littlesunday C. Jackson R. Meyer T. (2007). AM-111 protects against permanent hearing loss from impulse noise trauma. *Hear. Res.* 226 70–78. 10.1016/j.heares.2006.05.006 16839720

[B13] Creber N. J. Eastwood H. T. Hampson A. J. Tan J. O’Leary S. J. (2019). Adjuvant agents enhance round window membrane permeability to dexamethasone and modulate basal to apical cochlear gradients. *Eur. J. Pharm. Sci.* 126 69–81. 10.1016/j.ejps.2018.08.013 30107228

[B14] Cunningham L. L. Tucci D. L. (2017). Hearing Loss in Adults. *N. Engl. J. Med.* 377 2465–2473. 29262274 10.1056/NEJMra1616601 PMC6457651

[B15] Demir M. G. Altintoprak N. Aydin S. Kosemihal E. Basak K. (2015). Effect of transtympanic injection of melatonin on cisplatin-induced ototoxicity. *J. Int. Adv. Otol.* 11 202–206. 10.5152/iao.2015.1094 26915150

[B16] Ding S. Xie S. Chen W. Wen L. Wang J. Yang F. (2019). Is oval window transport a royal gate for nanoparticle delivery to vestibule in the inner ear? *Eur. J. Pharm. Sci.* 126 11–22. 10.1016/j.ejps.2018.02.031 29499347

[B17] Dormer N. H. Nelson-Brantley J. Staecker H. Berkland C. J. (2019). Evaluation of a transtympanic delivery system in Mus musculus for extended release steroids. *Eur. J. Pharm. Sci.* 126 3–10. 10.1016/j.ejps.2018.01.020 29329746 PMC6039288

[B18] El Kechai N. Mamelle E. Nguyen Y. Huang N. Nicolas V. Chaminade P. (2016). Hyaluronic acid liposomal gel sustains delivery of a corticoid to the inner ear. *J. Control Release* 226 248–257. 10.1016/j.jconrel.2016.02.013 26860286

[B19] Falcone P. H. Nieman K. M. Tribby A. C. Vogel R. M. Joy J. M. Moon J. R. (2019). The attention-enhancing effects of spearmint extract supplementation in healthy men and women: a randomized, double-blind, placebo-controlled, parallel trial. *Nutr. Res.* 64 24–38. 10.1016/j.nutres.2018.11.012 30802720

[B20] Fernandez R. Harrop-Jones A. Wang X. Dellamary L. LeBel C. Piu F. (2016). The sustained-exposure dexamethasone formulation OTO-104 offers effective protection against cisplatin-induced hearing loss. *Audiol. Neurootol.* 21 22–29. 10.1159/000441833 26789647

[B21] Fetoni A. R. Eramo S. L. M. Di Pino A. Rolesi R. Paciello F. Grassi C. (2018). The antioxidant effect of rosmarinic acid by different delivery routes in the animal model of noise-induced hearing loss. *Otol. Neurotol.* 39 378–386. 10.1097/MAO.0000000000001700 29424820

[B22] Franz P. Aharinejad S. Bock P. Firbas W. (1993). The cochlear glomeruli in the modiolus of the guinea pig. *Eur. Arch. Otorhinolaryngol.* 250 44–50. 8466749 10.1007/BF00176948

[B23] Gao G. Liu Y. Zhou C. H. Jiang P. Sun J. J. (2015). Solid lipid nanoparticles loaded with edaravone for inner ear protection after noise exposure. *Chin. Med. J.* 128 203–209. 10.4103/0366-6999.149202 25591563 PMC4837839

[B24] Gates G. A. Schmid P. Kujawa S. G. Nam B. D’Agostino R. (2000). Longitudinal threshold changes in older men with audiometric notches. *Hear. Res.* 141 220–228. 10.1016/s0378-5955(99)00223-3 10713509

[B25] Ghosh S. Sheth S. Sheehan K. Mukherjea D. Dhukhwa A. Borse V. (2018). The Endocannabinoid/Cannabinoid Receptor 2 System Protects Against Cisplatin-Induced Hearing Loss. *Front. Cell. Neurosci.* 12:271. 10.3389/fncel.2018.00271 30186120 PMC6110918

[B26] Goycoolea M. V. (1992). The round window membrane under normal and pathological conditions. *Acta Otolaryngol. Suppl.* 493 43–55. 1636422

[B27] Goycoolea M. V. Lundman L. (1997). Round window membrane. Structure function and permeability: a review. *Microsc. Res. Tech.* 36 201–211. 9080410 10.1002/(SICI)1097-0029(19970201)36:3<201::AID-JEMT8>3.0.CO;2-R

[B28] Goycoolea M. V. Muchow D. Schachern P. (1988). Experimental studies on round window structure: function and permeability. *Laryngoscope* 98(6 Pt 2 Suppl. 44), 1–20. 10.1288/00005537-198806001-00002 3287079

[B29] Gumrukcu S. S. Topaloglu I. Salturk Z. Tutar B. Atar Y. Berkiten G. (2018). Effects of intratympanic dexamethasone on noise-induced hearing loss: an experimental study. *Am. J. Otolaryngol.* 39 71–73. 10.1016/j.amjoto.2017.10.011 29110919

[B30] Han M. A. Back S. A. Kim H. L. Park S. Y. Yeo S. W. Park S. N. (2015). Therapeutic effect of dexamethasone for noise-induced hearing loss: systemic versus intratympanic injection in mice. *Otol. Neurotol.* 36 755–762. 10.1097/MAO.0000000000000759 25894725

[B31] Harrop-Jones A. Wang X. Fernandez R. Dellamary L. Ryan A. F. LeBel C. (2016). The sustained-exposure dexamethasone formulation OTO-104 offers effective protection against noise-induced hearing loss. *Audiol. Neurootol.* 21 12–21. 10.1159/000441814 26655654

[B32] Hazlitt R. A. Min J. Zuo J. (2018). Progress in the Development of Preventative Drugs for Cisplatin-Induced Hearing Loss. *J. Med. Chem.* 61 5512–5524. 10.1021/acs.jmedchem.7b01653 29361217 PMC6043375

[B33] Heinrich U. R. Strieth S. Schmidtmann I. Stauber R. Helling K. (2016). Dexamethasone prevents hearing loss by restoring glucocorticoid receptor expression in the guinea pig cochlea. *Laryngoscope* 126 E29–E34. 10.1002/lary.25345 25946598

[B34] Hellberg V. Wallin I. Ehrsson H. Laurell G. (2013). Cochlear pharmacokinetics of cisplatin: an in vivo study in the guinea pig. *Laryngoscope* 123 3172–3177. 10.1002/lary.24235 23754209

[B35] Hill G. W. Morest D. K. Parham K. (2008). Cisplatin-induced ototoxicity: effect of intratympanic dexamethasone injections. *Otol. Neurotol.* 29 1005–1011. 10.1097/MAO.0b013e31818599d5 18716567 PMC2720789

[B36] Hill K. Yuan H. Wang X. Sha S. H. (2016). Noise-induced loss of hair cells and cochlear synaptopathy are mediated by the activation of AMPK. *J. Neurosci.* 36 7497–7510. 10.1523/JNEUROSCI.0782-16.2016 27413159 PMC4945669

[B37] Juhn S. K. Meyerhoff W. L. Paparella M. M. (1981). Clinical application of middle ear effusion analyses. *Laryngoscope* 91 1012–1015. 7242181 10.1288/00005537-198106000-00023

[B38] Karasawa T. Steyger P. S. (2015). An integrated view of cisplatin-induced nephrotoxicity and ototoxicity. *Toxicol. Lett.* 237 219–227. 10.1016/j.toxlet.2015.06.012 26101797 PMC4516600

[B39] Kaur T. Borse V. Sheth S. Sheehan K. Ghosh S. Tupal S. (2016). Adenosine A1 receptor protects against cisplatin ototoxicity by suppressing the NOX3/STAT1 inflammatory pathway in the cochlea. *J. Neurosci.* 36 3962–3977. 10.1523/JNEUROSCI.3111-15.2016 27053204 PMC4821909

[B40] Kaur T. Mukherjea D. Sheehan K. Jajoo S. Rybak L. P. Ramkumar V. (2011). Short interfering RNA against STAT1 attenuates cisplatin-induced ototoxicity in the rat by suppressing inflammation. *Cell Death Dis.* 2:e180. 10.1038/cddis.2011.63 21776018 PMC3199718

[B41] Kayyali M. N. Wooltorton J. R. A. Ramsey A. J. Lin M. Chao T. N. Tsourkas A. (2018). A novel nanoparticle delivery system for targeted therapy of noise-induced hearing loss. *J. Control Release* 279 243–250. 10.1016/j.jconrel.2018.04.028 29673641 PMC6344933

[B42] King E. B. Salt A. N. Eastwood H. T. O’Leary S. J. (2011). Direct entry of gadolinium into the vestibule following intratympanic applications in Guinea pigs and the influence of cochlear implantation. *J. Assoc. Res. Otolaryngol.* 12 741–751. 10.1007/s10162-011-0280-5 21769689 PMC3214238

[B43] Korver K. D. Rybak L. P. Whitworth C. Campbell K. M. (2002). Round window application of D-methionine provides complete cisplatin otoprotection. *Otolaryngol. Head Neck Surg.* 126 683–689. 10.1067/mhn.2002.125299 12087338

[B44] Kros C. J. Steyger P. S. (2018). Aminoglycoside- and cisplatin-induced ototoxicity: mechanisms and otoprotective strategies^*^. *Cold Spring Harb. Perspect. Med.* a033548. 10.1101/cshperspect.a033548 30559254 PMC6579718

[B45] Kujawa S. G. Liberman M. C. (2009). Adding insult to injury: cochlear nerve degeneration after temporary noise-induced hearing loss. *J. Neurosci.* 29 14077–14085. 10.1523/jneurosci.2845-09.2009 19906956 PMC2812055

[B46] Kumar U. A. Ameenudin S. Sangamanatha A. V. (2012). Temporal and speech processing skills in normal hearing individuals exposed to occupational noise. *Noise Health* 14 100–105. 10.4103/1463-1741.97252 22718107

[B47] Kurabi A. Keithley E. M. Housley G. D. Ryan A. F. Wong A. C. (2017). Cellular mechanisms of noise-induced hearing loss. *Hear. Res.* 349 129–137. 10.1016/j.heares.2016.11.013 27916698 PMC6750278

[B48] Lavorgna M. Orlo E. Nugnes R. Piscitelli C. Russo C. Isidori M. (2019). Capsaicin in hot chili peppers: in vitro evaluation of its antiradical, antiproliferative and apoptotic activities. *Plant Foods Hum. Nutr.* 74 164–170. 10.1007/s11130-019-00722-0 30835044

[B49] Le Prell C. G. Clavier O. H. (2017). Effects of noise on speech recognition: challenges for communication by service members. *Hear. Res.* 349 76–89. 10.1016/j.heares.2016.10.004 27743882

[B50] Li G. Frenz D. A. Brahmblatt S. Feghali J. G. Ruben R. J. Berggren D. (2001). Round window membrane delivery of L-methionine provides protection from cisplatin ototoxicity without compromising chemotherapeutic efficacy. *Neurotoxicology* 22 163–176. 10.1016/s0161-813x(00)00010-3 11405249

[B51] Li L. Chao T. Brant J. O’Malley B. Jr. Tsourkas A. Li D. (2017). Advances in nano-based inner ear delivery systems for the treatment of sensorineural hearing loss. *Adv. Drug Deliv. Rev.* 108 2–12. 10.1016/j.addr.2016.01.004 26796230 PMC4940320

[B52] Li W. Hartsock J. J. Dai C. Salt A. N. (2018). Permeation enhancers for intratympanically-applied drugs studied using fluorescent dexamethasone as a marker. *Otol. Neurotol.* 39 639–647. 10.1097/MAO.0000000000001786 29649043 PMC5940507

[B53] Martin-Saldana S. Palao-Suay R. Aguilar M. R. Ramirez-Camacho R. San Roman J. (2017). Polymeric nanoparticles loaded with dexamethasone or alpha-tocopheryl succinate to prevent cisplatin-induced ototoxicity. *Acta Biomater.* 53 199–210. 10.1016/j.actbio.2017.02.019 28213099

[B54] Masterson E. A. Themann C. L. Calvert G. M. (2018). prevalence of hearing loss among noise-exposed workers within the health care and social assistance sector, 2003 to 2012. *J. Occup. Environ. Med.* 60 350–356. 10.1097/JOM.0000000000001214 29111986

[B55] More S. S. Akil O. Ianculescu A. G. Geier E. G. Lustig L. R. Giacomini K. M. (2010). Role of the copper transporter, CTR1, in platinum-induced ototoxicity. *J. Neurosci.* 30 9500–9509. 10.1523/JNEUROSCI.1544-10.2010 20631178 PMC2949060

[B56] Mukherjea D. Jajoo S. Sheehan K. Kaur T. Sheth S. Bunch J. (2011). NOX3 NADPH oxidase couples transient receptor potential vanilloid 1 to signal transducer and activator of transcription 1-mediated inflammation and hearing loss. *Antioxid. Redox Signal.* 14 999–1010. 10.1089/ars.2010.3497 20712533 PMC3043978

[B57] Mukherjea D. Jajoo S. Whitworth C. Bunch J. R. Turner J. G. Rybak L. P. (2008). Short interfering RNA against transient receptor potential vanilloid 1 attenuates cisplatin-induced hearing loss in the rat. *J. Neurosci.* 28 13056–13065. 10.1523/JNEUROSCI.1307-08.2008 19052196 PMC2865180

[B58] Munzel T. Sorensen M. Schmidt F. Schmidt E. Steven S. Kroller-Schon S. (2018). The adverse effects of environmental noise exposure on oxidative stress and cardiovascular risk. *Antioxid. Redox. Signal.* 28 873–908. 10.1089/ars.2017.7118 29350061 PMC5898791

[B59] Murphy D. Daniel S. J. (2011). Intratympanic dexamethasone to prevent cisplatin ototoxicity: a guinea pig model. *Otolaryngol Head Neck Surg.* 145 452–457. 10.1177/0194599811406673 21521888

[B60] Nader M. E. Theoret Y. Saliba I. (2010). The role of intratympanic lactate injection in the prevention of cisplatin-induced ototoxicity. *Laryngoscope* 120 1208–1213. 10.1002/lary.20892 20513041

[B61] Nelson D. I. Nelson R. Y. Concha-Barrientos M. Fingerhut M. (2005). The global burden of occupational noise-induced hearing loss. *Am. J. Ind. Med.* 48 446–458. 10.1002/ajim.20223 16299704

[B62] Nyberg S. Abbott N. J. Shi X. Steyger P. S. Dabdoub A. (2019). Delivery of therapeutics to the inner ear: the challenge of the blood-labyrinth barrier. *Sci. Transl. Med.* 11:eaao0935. 10.1126/scitranslmed.aao0935 30842313 PMC6488020

[B63] Ohinata Y. Miller J. M. Altschuler R. A. Schacht J. (2000). Intense noise induces formation of vasoactive lipid peroxidation products in the cochlea. *Brain Res.* 878 163–173. 10.1016/s0006-8993(00)02733-5 10996147

[B64] Ohlemiller K. K. (2008). Recent findings and emerging questions in cochlear noise injury. *Hear. Res.* 245 5–17. 10.1016/j.heares.2008.08.007 18790034 PMC2610263

[B65] Oishi N. Schacht J. (2011). Emerging treatments for noise-induced hearing loss. *Expert Opin. Emerg. Drugs* 16 235–245. 10.1517/14728214.2011.552427 21247358 PMC3102156

[B66] Ozdogan F. Ensari S. Cakir O. Ozcan K. M. Koseoglu S. Ozdas T. (2012). Investigation of the cochlear effects of intratympanic steroids administered following acoustic trauma. *Laryngoscope* 122 877–882. 10.1002/lary.23185 22374513

[B67] Özel H. E. Özdoǧan F. Gürgen S. G. Esen E. Genc̨ S. Selc̨uk A. (2016). Comparison of the protective effects of intratympanic dexamethasone and methylprednisolone against cisplatin-induced ototoxicity. *J. Laryngol. Otol.* 130, 225–234. 10.1017/S0022215115003473 26830667

[B68] Paciello F. Fetoni A. R. Rolesi R. Wright M. B. Grassi C. Troiani D. (2018). pioglitazone represents an effective therapeutic target in preventing oxidative/inflammatory cochlear damage induced by noise exposure. *Front. Pharmacol.* 9:1103. 10.3389/fphar.2018.01103 30349478 PMC6187064

[B69] Paksoy M. Ayduran E. Sanli A. Eken M. Aydin S. Oktay Z. A. (2011). The protective effects of intratympanic dexamethasone and vitamin E on cisplatin-induced ototoxicity are demonstrated in rats. *Med. Oncol.* 28 615–621. 10.1007/s12032-010-9477-4 20300971

[B70] Parham K. (2011). Can intratympanic dexamethasone protect against cisplatin ototoxicity in mice with age-related hearing loss? *Otolaryngol. Head Neck Surg.* 145 635–640. 10.1177/0194599811409304 21572077

[B71] Parhizkar N. Rybak L. (2003). “Round Window Application of the P53 Inhibitor Pifithrin-Alpha provides complete protection against Cisplatin Ototoxicity,” in *Proceedings of the 26th Annual Midwinter Research Meeting of The Association for Research in Otolaryngology*, Florida, FL.

[B72] Piu F. Wang X. Fernandez R. Dellamary L. Harrop A. Ye Q. (2011). OTO-104: a sustained-release dexamethasone hydrogel for the treatment of otic disorders. *Otol. Neurotol.* 32 171–179. 10.1097/MAO.0b013e3182009d29 21099726

[B73] Qi W. Ding D. Zhu H. Lu D. Wang Y. Ding J. (2014). Efficient siRNA transfection to the inner ear through the intact round window by a novel proteidic delivery technology in the chinchilla. *Gene. Ther.* 21 10–18. 10.1038/gt.2013.49 24108151 PMC3881030

[B74] Ramaswamy B. Roy S. Apolo A. B. Shapiro B. Depireux D. A. (2017). Magnetic nanoparticle mediated steroid delivery mitigates cisplatin induced hearing loss. *Front. Cell. Neurosci.* 11:268. 10.3389/fncel.2017.00268 28955202 PMC5601400

[B75] Salt A. N. Hirose K. (2018). Communication pathways to and from the inner ear and their contributions to drug delivery. *Hear. Res.* 362 25–37. 10.1016/j.heares.2017.12.010 29277248 PMC5911243

[B76] Salt A. N. Plontke S. K. (2009). Principles of local drug delivery to the inner ear. *Audiol. Neurootol.* 14 350–360. 10.1159/000241892 19923805 PMC2820328

[B77] Salt A. N. Plontke S. K. (2018). Pharmacokinetic principles in the inner ear: influence of drug properties on intratympanic applications. *Hear. Res.* 368 28–40. 10.1016/j.heares.2018.03.002 29551306 PMC6133771

[B78] Shafik A. G. Elkabarity R. H. Thabet M. T. Soliman N. B. Kalleny N. K. (2013). Effect of intratympanic dexamethasone administration on cisplatin-induced ototoxicity in adult guinea pigs. *Auris Nasus Larynx* 40 51–60. 10.1016/j.anl.2012.05.010 22884636

[B79] Sheehan K. Sheth S. Mukherjea D. Rybak L. P. Ramkumar V. (2018). Trans-tympanic drug delivery for the treatment of ototoxicity^*^. *J. Vis. Exp.* 56564. 10.3791/56564 29608150 PMC5931769

[B80] Sheth S. Mukherjea D. Rybak L. P. Ramkumar V. (2017). Mechanisms of cisplatin-induced ototoxicity and otoprotection. *Front. Cell. Neurosci.* 11:338. 10.3389/fncel.2017.00338 29163050 PMC5663723

[B81] Shi X. (2016). Pathophysiology of the cochlear intrastrial fluid-blood barrier (review). *Hear. Res.* 338 52–63. 10.1016/j.heares.2016.01.010 26802581 PMC5322264

[B82] Shih C. P. Chen H. C. Lin Y. C. Chen H. K. Wang H. Kuo C. Y. (2018). Middle-ear dexamethasone delivery via ultrasound microbubbles attenuates noise-induced hearing loss. *Laryngoscope* 10.1002/lary.27713 [Epub ahead of print]. 30588634

[B83] Shin Y. S. Song S. J. Kang S. U. Hwang H. S. Choi J. W. Lee B. H. (2013). A novel synthetic compound, 3-amino-3-(4-fluoro-phenyl)-1H-quinoline-2,4-dione, inhibits cisplatin-induced hearing loss by the suppression of reactive oxygen species: in vitro and in vivo study. *Neuroscience* 232 1–12. 10.1016/j.neuroscience.2012.12.008 23246618

[B84] Sly D. J. Campbell L. Uschakov A. Saief S. T. Lam M. O’Leary S. J. (2016). Applying neurotrophins to the round window rescues auditory function and reduces inner hair cell synaptopathy after noise-induced hearing loss. *Otol. Neurotol.* 37 1223–1230. 10.1097/MAO.0000000000001191 27631825

[B85] Sun C. Wang X. Zheng Z. Chen D. Wang X. Shi F. (2015). A single dose of dexamethasone encapsulated in polyethylene glycol-coated polylactic acid nanoparticles attenuates cisplatin-induced hearing loss following round window membrane administration. *Int. J. Nanomed.* 10 3567–3579. 10.2147/IJN.S77912 25999718 PMC4437605

[B86] Suzuki J. Corfas G. Liberman M. C. (2016). Round-window delivery of neurotrophin 3 regenerates cochlear synapses after acoustic overexposure. *Sci. Rep.* 6:24907. 10.1038/srep24907 27108594 PMC4842978

[B87] Tanaka K. Motomura S. (1981). Permeability of the labyrinthine windows in guinea pigs. *Arch. Otorhinolaryngol.* 233 67–73. 6976164 10.1007/BF00464276

[B88] Teitz T. Fang J. Goktug A. N. Bonga J. D. Diao S. Hazlitt R. A. (2018). CDK2 inhibitors as candidate therapeutics for cisplatin- and noise-induced hearing loss. *J. Exp. Med.* 215 1187–1203. 10.1084/jem.20172246 29514916 PMC5881471

[B89] Teranishi M. A. Nakashima T. (2003). Effects of trolox, locally applied on round windows, on cisplatin-induced ototoxicity in guinea pigs. *Int. J. Pediatr. Otorhinolaryngol.* 67 133–139. 10.1016/s0165-5876(02)00353-1 12623149

[B90] Wang J. Ruel J. Ladrech S. Bonny C. van de Water T. R. Puel J. L. (2007). Inhibition of the c-Jun N-terminal kinase-mediated mitochondrial cell death pathway restores auditory function in sound-exposed animals. *Mol. Pharmacol.* 71 654–666. 10.1124/mol.106.028936 17132689

[B91] Wang X. Chen Y. Tao Y. Gao Y. Yu D. Wu H. (2018). A666-conjugated nanoparticles target prestin of outer hair cells preventing cisplatin-induced hearing loss. *Int. J. Nanomed.* 13 7517–7531. 10.2147/IJN.S170130 30532536 PMC6241721

[B92] Wimmer C. Mees K. Stumpf P. Welsch U. Reichel O. Suckfull M. (2004). Round window application of D-methionine, sodium thiosulfate, brain-derived neurotrophic factor, and fibroblast growth factor-2 in cisplatin-induced ototoxicity. *Otol. Neurotol.* 25 33–40. 10.1097/00129492-200401000-00007 14724489

[B93] Xiong H. Long H. Pan S. Lai R. Wang X. Zhu Y. (2019). Inhibition of histone methyltransferase g9a attenuates noise-induced cochlear synaptopathy and hearing loss. *J. Assoc. Res. Otolaryngol.* 20 217–232. 10.1007/s10162-019-00714-6 30710318 PMC6513956

[B94] Yamane H. Nakai Y. Takayama M. Iguchi H. Nakagawa T. Kojima A. (1995). Appearance of free radicals in the guinea pig inner ear after noise-induced acoustic trauma. *Eur. Arch. Otorhinolaryngol.* 252 504–508. 10.1007/bf02114761 8719596

[B95] Zhang L. Xu Y. Cao W. Xie S. Wen L. Chen G. (2018). Understanding the translocation mechanism of PLGA nanoparticles across round window membrane into the inner ear: a guideline for inner ear drug delivery based on nanomedicine. *Int. J. Nanomed.* 13 479–492. 10.2147/IJN.S154968 29403277 PMC5784583

[B96] Zhang M. Liu W. Ding D. Salvi R. (2003). Pifithrin-alpha suppresses p53 and protects cochlear and vestibular hair cells from cisplatin-induced apoptosis. *Neuroscience* 120 191–205. 10.1016/s0306-4522(03)00286-0 12849752

[B97] Zhou Y. Zheng H. Shen X. Zhang Q. Yang M. (2009). Intratympanic administration of methylprednisolone reduces impact of experimental intensive impulse noise trauma on hearing. *Acta Otolaryngol.* 129 602–607. 10.1080/00016480802342424 18815936

[B98] Zou J. Pyykko I. Hyttinen J. (2016). Inner ear barriers to nanomedicine-augmented drug delivery and imaging. *J. Otol.* 11 165–177. 10.1016/j.joto.2016.11.002 29937826 PMC6002620

